# The Role of CT and MR Imaging in Stereotactic Body Radiotherapy of the Spine: From Patient Selection and Treatment Planning to Post-Treatment Monitoring

**DOI:** 10.3390/cancers16213692

**Published:** 2024-10-31

**Authors:** Javid Azadbakht, Amy Condos, David Haynor, Wende N. Gibbs, Pejman Jabehdar Maralani, Arjun Sahgal, Samuel T. Chao, Matthew C. Foote, John Suh, Eric L. Chang, Matthias Guckenberger, Mahmud Mossa-Basha, Simon S. Lo

**Affiliations:** 1 Department of Radiology, University of Washington School of Medicine, Seattle, WA 98195, USA; 2 Department of Radiology, Barrow Neurological Institute, Phoenix, AZ 85013, USA; 3 Department of Medical Imaging, Sunnybrook Health Sciences Centre, University of Toronto, Toronto, ON M4N 3M5, Canada; 4 Department of Radiation Oncology, Sunnybrook Research Institute, Toronto, ON M4N 3M5, Canada; 5 Department of Radiation Oncology, Case Western Reserve University, Cleveland, OH 44106, USA; 6 Department of Radiation Oncology, Princess Alexandra Hospital, University of Queensland, Brisbane, QLD 4102, Australia; 7 Department of Radiation Oncology, Norris Comprehensive Cancer Center, Keck School of Medicine, University of Southern California, Los Angeles, CA 90089, USA; 8 Department of Radiation Oncology, University Hospital Zürich and University of Zürich, 8091 Zürich, Switzerland; 9 Department of Radiation Oncology, University of Washington School of Medicine, Seattle, WA 98195, USA

**Keywords:** stereotactic body radiotherapy, spine, metastasis, MRI, diffusion imaging, perfusion imaging

## Abstract

Spine metastases (SMs) are common, arising in 70% of the cases of the most prevalent malignancies in males (prostate cancer) and females (breast cancer). Stereotactic body radiotherapy, or SBRT, has been incorporated into clinical treatment algorithms over the past decade. SBRT has shown promising rates of local control for oligometastatic spinal lesions with low radiation dose to adjacent critical tissues, particularly the spinal cord. Imaging is critically important in SBRT planning, guidance, and response monitoring. This paper reviews the roles of imaging in spine SBRT, including conventional and advanced imaging approaches for SM detection, treatment planning, and post-SBRT follow-up.

## Simple Summary

The spine is the most common site of osseous metastatic involvement. Advances in diagnosing and treating spinal metastasis have improved patient survival and quality of life. There is now a strong focus on customized imaging to enhance treatment planning for tumor ablation and reduce treatment-related complications. This review discusses imaging protocols and findings in the pre- and post-SBRT setting and the imaging features differentiating expected post-SBRT treatment changes from LF.

## 1. Introduction

The spine is the third most common site of metastatic disease after lung and liver [1]. Spine metastases (SMs) most commonly originate from lung, prostate, and breast cancers [2,3,4,5], although malignancies of any organ may involve the spine. An abundance of red marrow in the vertebral bodies [6] and characteristics of the Batson venous plexus [7] contribute to the high rate of hematogenous spread, and help explain why the spine is the most common site of osseous metastatic involvement, with a prevalence of 70% in those with breast and prostate malignancies according to post-mortem studies [8].

SMs can cause debilitating axial pain and impair the quality of life and performance status. Pain can arise from pathological vertebral compression fractures (VCFs) and/or compressive radiculopathy/myelopathy from epidural tumor spread, among other possible causes. Neurological dysfunction may ensue, further impairing performance status. Metastatic epidural spinal cord compression (MESCC) can explain symptoms in up to 20% of SMs, and can be the cause of an oncologic emergency needing emergent treatment in the presence of neurologic symptoms [2,3,9,10,11].

The treatment options available for SMs are radiotherapy (RT) with various radiation methods and dosing/fractioning regimens, surgery (open or minimally invasive spine surgery), neurointerventional procedures, and combination approaches, specifically decompressive surgery and stabilization followed by targeted radiotherapy.

SBRT has become a major component of treatment paradigms for malignancies (and even some benign lesions) around or within neural tissues, including SMs, with almost 50% of radiation oncologists incorporating it into their daily practice [12]. SBRT provides targeted focal treatment, limiting radiation exposure to neural elements, and thus permitting higher ablative doses to metastases. In the modern era, where therapeutic goals have shifted from merely managing symptoms to reducing post-treatment local failure (LF) rates, SBRT is a routine component in both definitive and adjuvant therapy strategies for neuro-oncologic conditions [13]. Conventional and advanced spine imaging is integral to SBRT planning, increases its therapeutic potential, and reduces the risk of post-SBRT morbidity. Imaging is also central to initial disease detection, patient selection for SBRT, post-SBRT response assessment, and longitudinal evaluation for recurrence [9].

With the ongoing advancements in the diagnosis and treatment of SMs, along with enhanced patient survival and quality of life, there is a clinical emphasis on tailored imaging to optimize treatment planning for tumor ablation while minimizing treatment-induced morbidities. This review article outlines conventional and advanced CT and MRI approaches for SBRT pre-treatment, treatment planning, and post-treatment evaluation for ordering providers, including oncologists, and future imaging directions that may better inform treatment decision making.

## 2. Conventional Radiotherapy Versus Stereotactic Body Radiotherapy

RT is beneficial in most SMs cases with few exceptions, and a combination of surgery and RT provides better clinical outcomes compared to RT alone in selected patients with MESCC [14].

SBRT is an established treatment for SMs, with a growing body of evidence supporting its effectiveness in local symptom palliation and disease control [15,16,17,18,19,20], and in patients with oligoprogressive disease [21].

SBRT utilizes advanced RT techniques to deliver ablative doses of radiation to the target tissue while minimizing radiation dose to the surrounding normal organs [22]. SBRT has demonstrated promising results as primary treatment (de novo), the treatment of external-beam radiotherapy (cEBRT) failures, post-surgery residual tumor treatment, and benign spinal tumor management, with or without the prior histopathological characterization of the tumor [23,24,25]. SBRT has a local pain response rate of 74.3% and a high durable local control rate of 80–95%, 70–100%, and 66–93% in primary, post-operative, and re-irradiation treatments, respectively [26,27,28,29,30]. Although SBRT provides better local control compared to EBRT, post-SBRT local recurrences still occur, mostly in the epidural space, especially when the pre-SBRT Epidural Spinal Cord Compression Classification (ESCC) score (also known as the Bilsky scale) is high, with an LF rate of 5%, 19%, and 30% in cases with no epidural disease (grade 0), epidural disease with low (grade 1a–c), or high (grade 2/3) ESCC scores, respectively [31,32]. These higher LF rates may be due to the underdosing of epidural disease because of dosimetric constraints and/or reflect the biologic aggressiveness of the metastases [33].

cEBRT typically delivers up to 5 Gy per fraction [25], which is considered hypofractionated, while SBRT, delivered in 1 to 5 fractions, provides higher biologically effective doses (BEDs) to a more precisely delineated target area. In addition to the BED, the distinctive geometry and steep dose gradients in SBRT play a significant role in its therapeutic effects compared to conventional radiotherapy. For these reasons, SBRT is highly effective in tumor ablation, but is also more challenging to plan with target proximity to organs at risk (OARs) (e.g., spinal cord, nerve roots and plexuses, and gastrointestinal tract). Although steep dose gradients spare the off-field areas, positional variations as small as one millimeter may result in adverse effects in normal critical tissues [34].

EBRT remains the preferred mode of irradiation in patients with poor overall performance status and prognosis (such as those with symptomatic systemic metastatic disease involving critical tissues, Karnofsky performance status ≤ 40, or a life expectancy of <2 months).

## 3. Imaging Role in Patient Selection for SBRT

After evaluating a patient’s prognosis and disease burden, metastatic lesions that are candidates for RT can be evaluated with respect to Mechanical Stability, Neurological Risk, Oncological Parameters, and Preferred Treatment (MNOP algorithm [9]).

To assess stability, the SINS (Spinal Neoplastic Instability Score) criteria are used. SINS entails six imaging and clinical features (pain, location of the lesion, density of the bone lesion (lytic/blastic), spine alignment, the presence of vertebral body collapse or extent of involvement, and posterior element involvement), and is scored with a range of 0-18 (Table 1). For SINS of 12 or above, spine surgeons should be consulted for the need for stabilizing intervention prior to SBRT. The defined role of surgery in SINS intermediate is still an active area of investigation but a consultation is advised [35].

**Figure 1:**
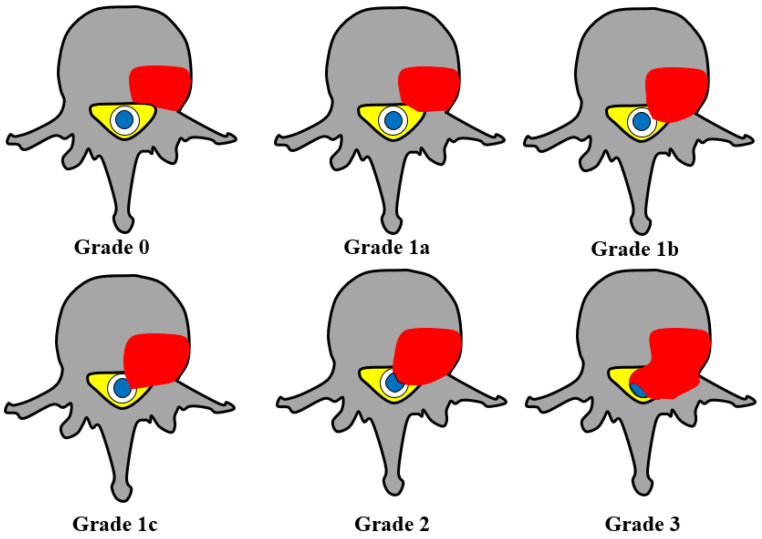
A schematic presentation of ESCC scores. Grade 0, no gross epidural disease; Grade 1a, tumor spread to epidural space with no obvious thecal sac indentation; Grade 1b, thecal sac indentation without spinal cord abutment; Grade 1c, spinal cord abutment without compression; Grade 2, spinal cord compression without subarachnoid space obliteration; and Grade 3, spinal cord compression with complete subarachnoid space obliteration.

Neurologic risk is assessed, in part, using the ESCC score. This is a measure of epidural metastatic disease resulting in spinal cord encroachment and helps guide whether SBRT can be used as a primary treatment with limited risk for spinal cord injury. The ESCC score is an imaging-based six-category scale [22,36]. Figure 1 displays a schematic depiction and description of the ESCC scores.

**Figure 2:**
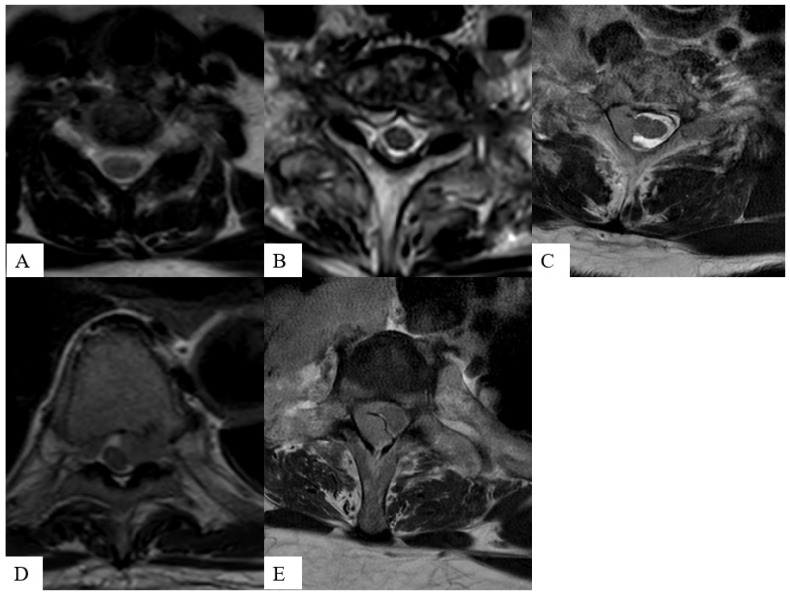
Axial T2-weighted images demonstrate the increasing severity of the epidural space involvement in patients with spinal metastasis from images (**A**) to (**E**): ESCC grade 1a shows tumor spreading to epidural space without indenting the thecal sac (**A**); grade 1b shows tumor causing indentation of the thecal sac (**B**); grade 1c shows tumor abutting but not compressing the spinal cord (**C**); grade 2 shows tumor compressing the spinal cord (**D**); and grade 3 shows tumor completely obliterating the subarachnoid space (**E**).

The ESCC score is assessed on axial T2-weighted images, which demonstrate superior inter- and intra-rater agreement when compared to T1-weighted and T1-weighted post-contrast acquisitions when used by a panel of expert reviewers [36] (examples shown in Figure 2). When combined with clinical and histopathological features, the ESCC score provides useful guidance for SMs therapy. ESCC scores of ≤1b are optimal candidates for primary SBRT. The optimal management of ESCC 1c-3 is case-specific, while spines with ESCC scores of 2 or 3 may undergo surgical intervention prior to SBRT to avoid neural complications, contingent on tumor radiosensitivity [10,36,37,38]. Spinal cord tolerance must be respected with underdosing as needed to achieve the constraints [39].

The principal oncological parameters are tumor histology and radiosensitivity, while tumor vascularity can be assessed to find out if pre-surgical embolization should be pursued. Exploring baseline data on tumor microvascularity and perfusion could pave the way for investigating vascularity as a potential marker for recurrence over time.

For malignant histologies that are highly sensitive to RT and/or systemic therapies (lymphoma and myeloma), cEBRT is recommended. On the other hand, radioresistant tumor types (sarcoma, melanoma, and renal cell carcinoma) may greatly benefit from ablative SBRT doses (especially when tumor burden is limited) [32,40,41]. Patients who cannot undergo MRI for pre-SBRT planning or RT guidance are not good candidates for SBRT [23].

In summary, SM treatment with SBRT is a complex treatment, and so it is preferred for patients with good overall prognosis: limited systemic disease (oligometastatic disease), small site of spine involvement (limited to 1–3 contiguous spinal/paraspinal levels), limited epidural disease (as graded by the ESCC score), and relatively stable spinal column (as defined by the Spinal Instability Neoplastic Score [SINS] criteria) [42].

In our clinical practice, radiologists report the SINS and ESCC scores for all SM cross-sectional exams to inform urgency and approaches to patient treatment. For SINS, we generally report the level with the highest SINS, if there are multiple levels with spinal involvement, while for ESCC, every level in which there is epidural disease is reported.

## 4. Pre-SBRT Imaging

Treatment planning with pre-SBRT imaging and tumor delineation is pivotal to confine the ablative doses of radiation closely to the target volume and prevent the inadvertent overdosing of normal tissues. This requires precise and reproducible positioning. For example, spinal cord overdosage can occur with very small patient bulk movements (as small as 1mm [43]) or if an epidural tumor touches or compresses the spinal cord. Radiation-induced myelitis, although as rare as 0.4%, is one of the most feared and debilitating late post-SBRT complications [34,44]. Contouring brachial or lumbosacral plexuses prior to SBRT is not routinely performed, despite the fact that these are well-studied OARs [45,46]. While radiation-induced plexopathy is rare in spine SBRT, there may be instances where the recommended dosimetric constraints [47] cannot be perfectly adhered to, leading to the inclusion of the spinal nerve roots within the clinical target volume (CTV) and their exposure to radiation [45].

Allowable delays between pre-SBRT imaging and treatment differ from center to center and depend on imaging and treatment access and institutional preferences. At our institution, patients are referred for pre-SBRT imaging within one week of SBRT, and preferably as close as possible to the treatment date/time.

### 4.1. Conventional Imaging

The SPIne response assessment in Neuro-Oncology (SPINO) group suggests utilizing both CT and conventional MRI in pre-SBRT imaging [48].

CT is superior in delineating osseous remodeling or erosion by tumor and is needed for SINS scoring, but is of limited accuracy in delineating soft tissue and bone marrow tumor infiltration. SPINO consensus guidelines recommend a slice thickness of at least ≤2 mm, preferably ≤1 mm, for treatment planning CT [48,49]. Conventional CT can be complemented with MRI or PET/CT to define soft tissue lesions and adjacent normal tissues and facilitate optimal treatment planning [49,50]. The fusing of multimodality image datasets can provide improved lesion assessment, but hybrid images must be reviewed to ensure coregistration accuracy. Any coregistration error in fused images will increase the risk of morbidity due to inadvertent dosing to critical structures [23].

MRI is the gold standard imaging modality in detecting and characterizing SMs, and is recommended for pre-SBRT imaging by the SPINO group and International Spine Radiosurgery Consortium [25,48,49,51]. Both in the cases of single isolated SMs or in those with multiple SMs, full spine MRI is recommended, as finding additional spinal lesions is not an uncommon finding. When thin-section volumetric MR images are taken, MRI fusion with CT should be performed [48,49]. Both 1.5-Tesla and 3-Tesla (3-T) MRI scanners are routinely used in clinical practice [52]. The 3-T scanners can facilitate faster acquisition with thinner slices. Three-dimensional MRI techniques can further shorten the scan time and improve image resolution. The use of 3-T scanning may lead to increased susceptibility artifacts (a major issue in patients with fixation devices), chemical shift artifacts (which may distort bone interfaces), and longer relaxation times (which can decrease contrast on T1-weighted images).

As per the International Spine Research Consortium, the recommended MR sequences for SBRT planning are volumetric non-contrast T1-weighted (T1W) and T2-weighted (T2W) imaging [48]. Sagittal T1W and STIR sequences are usually the most informative unenhanced MR sequences for SM detection. T1W imaging directly assesses marrow fat replacement by SMs earlier than CT which visualizes bone destruction [53,54]. The presence of iso- or hypointense lesions on T1 images, relative to adjacent paraspinal muscles or non-dehydrated intervertebral disks, predicts SMs with high sensitivity (94–100%) and specificity (92–94%) [55]. T1 imaging may not be able to differentiate between diffuse metastatic disease or red marrow conversion induced by granulocyte colony-stimulating factor [49,56]. STIR image may show fluid signal when marrow is infiltrated, and offers a more accurate measurement of true SMs size and better delineation between tumor and peritumoral edema compared to T1W imaging [57]. STIR may miss highly sclerotic lesions that can have no or little associated signal abnormality, and T1W images are more reliable in sclerotic SMs [58].

The axial T2-weighted image is optimal for spinal cord delineation and ESCC scoring [36,57]. Some centers also include axial T1W and STIR sequences [58]. Post-contrast T1W sequences, if performed, should be fat-suppressed in order to differentiate post-contrast enhancement from epidural and marrow fat signals [59]. Post-contrast images detect enhancing SMs; delineate epidural, paraspinal, and foraminal tumor spread; and leptomeningeal or intramedullary metastatic involvement [60], and can guide tissue biopsy.

### 4.2. Three-Dimensional (3-D) MR Imaging

Isotropic volumetric MRI acquisitions facilitate fusion with CT for pre-SBRT planning. With isotropic volumetric acquisition, multiplanar reconstructions can be carried out without image quality degradation [22]. Yamanaka et al. fused 3-D lumbar MRI with 3-D CT in two patients with spondyloarthropathy and complex anatomy [61]. They concluded that hybrid 3-D MRI/CT can offer a detailed anatomy of bony structures and neural elements, and their relationship, as well as delineating epidural space boundaries (such as ligamentum flavum) at a reasonable resolution with multiplanar reconstructions. This capability of 3-D imaging and the possibility of its coregistration with CT can provide an excellent depiction for SBRT treatment planning. The long acquisition times required for 3-D imaging are barriers to clinical adoption. Recent 3-D MRI acceleration methods, such as compressed sensing [CS] and AI de-noising reconstructions, however, have shown the image quality advantages of 3-D over 2-D MRI for SBRT planning [62]. Registration errors can be mitigated using various registration strategies. In the flexible spine, registration models that include vertebral dynamics, encompassing both rigid components (individual or fused vertebral bodies) and flexible segments, offer improved registration that preserves anatomic integrity. Some registration strategies, such as surface-matching, landmark, and fiducial-based registration, require user interaction which can increase registration errors, while intra-operative imaging-based registration offers a smaller mean target registration error [63,64,65,66].

## 5. Treatment Planning Imaging

SPINO advises incorporating both CT and conventional MRI in treatment planning imaging for SBRT to ensure a comprehensive assessment of bone and soft tissue involvement [48]. To keep radiation to critical tissues at a tolerable level, OARs should be accurately delineated. The gross tumor volume (GTV) consists of the metastatic tumor and its epidural/paraspinal extensions, as they appear on MRI. The clinical target volume (CTV) includes the whole anatomic compartment containing the GTV and the immediately adjacent anatomic compartments to include potentially viable microscopic invasion [49,51,67]. Including a diseased vertebral body in its entirety in the CTV has been associated with better post-SBRT local control in SMs [67]. The planning target volume (PTV) adds an additional rim of normal tissue to the CTV to compensate for motion, including cardiorespiratory and patient bulk motion [68]. Different centers consider a range of 0 to 3 mm expansion for PTV [51].

In post-operative patients with tumor recurrence or residue, CTV should include the full area of pre-treatment bony and epidural tumor involvement, as well as the surrounding bony structures at risk of microscopic tumor infiltration. Additionally, post-operative patients who have undergone tumor removal or treatment and subsequently developed a recurrence should have the CTV that includes the regions of pre-operative tumor involvement [69]. The delineation of the CTV in the post-operative setting can be guided according to the pattern of epidural failure. Chan et al. [70] recommended a horse-shoe-shaped CTV (excluding epidural space associated with spinous process) for cases with anterior but not posterior epidural failure in both pre- and post-operative MRI, and a donut-shaped CTV (covering the entire epidural space circumferentially and prophylactically) for those with anterior and posterior epidural failure.

SBRT requires accurate patient and target volume positioning and localization to ensure complete CTV coverage and decrease radiation to OARs. Patient bulk motion can be addressed by expanding the PTV to 1.5 mm beyond the CTV and using near-rigid body immobilization devices [68], such as BodyFIX^®^ (Elekta AB, Stockholm, Sweden), Civco Body Pro-Lok devices (Civco Medical Solutions; Orange City, IA, USA), and the Qfix Arm Shuttle™ (Qfix, Avondale, PA, USA). Markerless three-dimensional position tracing approaches have also been described in the literature [71,72,73].

For SBRT treatment planning, at our institution, we use an advanced imaging protocol to provide high-resolution anatomic as well as advanced physiological imaging. We incorporate 3-D T1-weighted pre- and post-contrast fat-suppressed, and 3-D T2-weighted gradient-recalled echo sequences. These sequences are scanned in a sagittal plane for faster acquisitions, but can be reconstructed in any plane due to isotropic spatial resolution, permitting registration with pre-treatment CT and SBRT planning software. In addition, we acquire dynamic 3-D balanced fast field echo (BFFE) to monitor spinal cord positioning throughout the cardiac cycle (discussed below) for better treatment targeting and to reduce the potential risk of cord dosing. We also perform the dynamic contrast-enhanced (DCE) perfusion of the spine (discussed below) to identify active versus quiescent metastatic disease and lesions that may not easily be detected on conventional spine MRI. We also perform the non-contrast CT of the targeted spine regions, which we also input into the treatment software for anatomic delineation and registration with MRI. In patients with substantial hardware that limits MR image quality, we perform CT myelography to delineate subarachnoid space, spinal cord, and epidural tumor, in place of both MRI and anatomic CT acquisitions.

## 6. MR-LINAC

The integration of MR imaging with modern linear accelerators (MR-LINAC) enables the real-time monitoring of anatomic and physiologic changes in tumors and surrounding tissues for more precise MRI-guided radiotherapy. Its ability to track anatomical changes during treatment reduces uncertainties caused by movement, leading to smaller planning margins, control of healthy tissue irradiation, and reduced toxicity, enhancing SBRT safety and efficacy [74,75].

Currently, two MR-LINAC systems are in use globally: Elekta’s Unity and ViewRay’s MRIdian, both employing coplanar static IMRT fields. Unity uses a 1.5 Tesla magnet with a 500 MU/min dose rate, featuring a small gap in the gradient coil and magnet winding. MRIdian, with a 0.35 Tesla split magnet, offers reduced magnetic field interaction and a higher dose rate of 650 MU/min [76,77]. Maximum gantry rotation speeds are 6 rpm for Unity and 0.5 rpm for MRIdian, and neither system has collimator rotation [74,78]. MRIdian uses a steady-state free-precession (SSFP) sequence for high-resolution planning and delivery, but can also acquire T1, T2, and DWI sequences. Unity offers a broader range of pulse sequences. Both systems use imaging for intra-treatment gating, automatically stopping the radiation beam if volumes exceed the set margins [74].

Adaptive radiotherapy with MR-LINAC is performed through two techniques from Elekta’s Unity: Adapt to Shape (ATS) and Adapt to Position (ATP). ATS is a more comprehensive approach that requires recontouring and replanning while the patient is on the treatment table, providing a more robust but time-consuming method [79]. ATP involves a virtual isocenter adjustment without recontouring, typically used in cases with minimal interfraction changes. In ATS-Lite, the target remains stable between sessions, with adjustments focused on relevant OARs [79].

## 7. Post-SBRT Follow-Up Imaging

Post-treatment imaging is important, particularly with regard to epidural involvement and resultant MESCC. Post-SBRT tumor progression in patients with sarcoma metastases to the spine, for example, was reported to occur at levels distant from the initial index lesion, supporting the importance of including the full spine in post-SBRT imaging [80].

The SPINO group has provided standardized consensus criteria for symptom-based response assessment in post-SBRT imaging [48]. The suggested protocol is MRI for post-SBRT response assessment every 2–3 months after radiation for the first year followed by subsequent MRIs at 3–6-month intervals in cases with no relevant new or progressing neurological deficit. Patients with neurological symptoms should be imaged immediately. The group also advocates the use of clear definitions of local control (no tumor progression on at least two MRI scans more than 6 weeks apart) and local progression (tumor enlargement or new tumor in the epidural space and/or recent neurological deterioration in the setting of epidural disease).

The SPINO group also highlighted the importance of considering osseous pseudoprogression (OPP) and osteonecrosis when evaluating post-SBRT studies. OPP and osteonecrosis can be ruled out by tissue sampling if they cannot be unequivocally differentiated from treatment failure on imaging, because salvage therapies may be associated with post-treatment morbidity. When clear differentiation cannot be achieved, recent research suggests that short-term imaging follow-up could provide a non-invasive alternative to biopsy for differentiation, though further verification is needed [81].

CT is mainly considered complementary to MRI in post-SBRT longitudinal follow-up with similar considerations as in the pre-SBRT setting. CT demonstrates bony anatomy and boundaries and identifies osseous changes, including lytic and blastic transformation with high accuracy [82]; however, CT is limited for soft tissue assessment, and thus should be fused with MRI for recurrent/residual soft tissue tumor delineation or monitoring response assessment [83]. CT can show re-ossification that typically takes place after successful RT and is well correlated with the alleviation of symptoms [84,85,86]. Vassilios et al. observed a 3-fold, 2-fold, and 1.5-fold rise in mean bone density for lytic, mixed, and osteoblastic bone metastases, respectively, compared to base line CT images following a combined treatment regimen of RT and ibandronate after 10 months [85]. The observed increase in bone density was hypothesized to be due to reduced osteoclast activity following RT, which was inversely associated with bone pain in the same study cohort.

MRI remains the primary imaging modality for post-SBRT evaluation, with the highest inter-rater agreement, as for pre-SBRT assessment [87]. Post-SBRT follow-up MRI should include sagittal T1W and axial T2W sequences, with slice thickness ≤ 3 mm for GTV/CTV delineation. The routine use of post-gadolinium MRI sequences with fat-suppression is a subject of controversy due to its tendency to enhance both normal bone marrow and SMs in spinal bone segments, thereby complicating differentiation. However, it enhances the visibility of paraspinal and epidural disease as well as leptomeningeal tumor spread [48]. STIR sequence can also be incorporated as it can increase the detectability of tumor recurrence [88].

Post-SBRT MRI detects SMs with excellent sensitivity and specificity, and can differentiate benign versus malignant VCFs with meaningfully higher accuracy than CT [88]. It also diagnoses epidural soft tissue infiltration with acceptable sensitivity. Post-treatment epidural tumor shrinkage or stability has been correlated to good SBRT response [89]. In small studies evaluating imaging differences between post-SBRT treatment-induced marrow signal changes versus LF, epidural T2 hyperintensity has shown divergent findings. One study showed T2 hyperintensity associated with treatment-related changes [89] while a separate study indicated an association with LF [90]. Vassilios et al. [84] examined 17 metastatic bone lesions in seven patients with post-RT clinical improvement, and reported a significant decrease in T1 signal intensity on both pre- and post-contrast MRI scans 3 months into the treatment.

If metallic hardware is placed in the spine, susceptibility artifacts from magnetic field inhomogeneity will distort the images and limit the visualization of the adjacent structures, including the spinal canal, spinal cord, and bony column. Metal artifact reduction techniques, vendor-specific sequences, lower field-strength imaging, and low TE sequences can reduce but not completely eliminate the limitations; CT myelography is the preferred imaging modality for spinal cord delineation in patients with spinal metallic hardware if additional information is required.

## 8. Advanced MR Imaging

Perfusion and diffusion-weighted MRI are the most extensively researched advanced imaging modalities in spine SBRT. Positron Emission Tomography (PET) is outside the scope of this review which primarily focuses on advanced MR techniques.

Although MRI is the mainstay for imaging SMs, the morphological characteristics of the lesions provide limited information on tumor pathophysiological characteristics and viability. Advanced imaging, when fused with conventional imaging for SBRT planning, can lead to a 25% reduction in critical structure irradiation [91]. Standard CT and MRI provide anatomic evaluation, while advanced imaging can better depict tumor viability and/or pathophysiology. Multiparametric post-SBRT MRI, including conventional and advanced MRI, can be advantageous to assess treatment response and capture treatment failure [92]. Advanced imaging allows for the improved identification of tumor cellularity and vascularity, aiding not only in treatment guidance but also in distinguishing between benign and malignant tumors in the pre-treatment phase or benign and malignant VCF/epidural lesion in the post-treatment phase [59].

### 8.1. DCE Perfusion Imaging

Dynamic contrast-enhanced (DCE) MR perfusion has shown promise in spine metastasis imaging. MR perfusion can reveal the hemodynamic and microvascular architecture of the tissue which are not assessable with conventional MRI or T2* dynamic susceptibility contrast perfusion [93]. T1 DCE perfusion employs a dynamic gradient-recalled echo sequence to track temporal changes in tissue gadolinium concentration. Optimal temporal resolution is essential for accurate tissue contrast kinetics [94].

**Figure 3:**
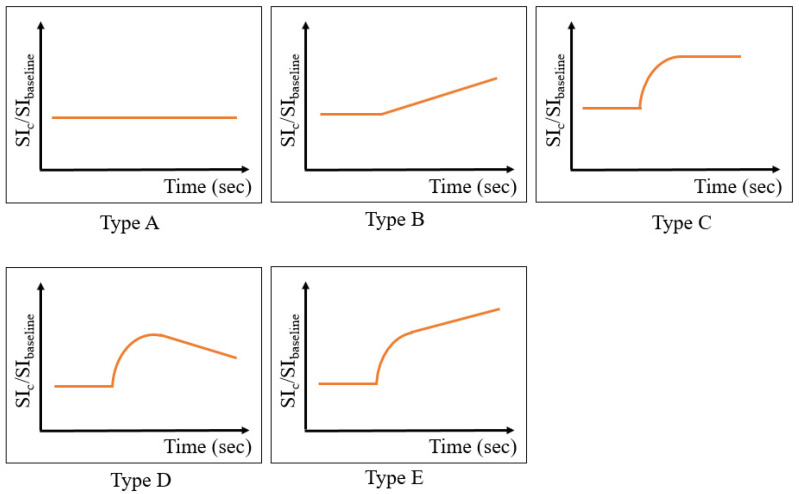
Different types of time–intensity curves plotting the relative enhancement percentage over time: type A, no perceptible enhancement; type B, gradual rise in intensity; type C, rapid wash-in then plateau; type D, rapid wash-in then wash-out; and type E, rapid wash-in then slower intensity rise. SI_C_, signal intensity after contrast injection; SI_baseline_, mean signal intensity of the baseline. A type D curve is suggestive of malignancy, while benign compression fractures show type E curves.

Vascular density, blood flow dynamics, and extravascular compartment characteristics are some factors that impact enhancement patterns and gadolinium sequestration in tissues and are discriminating features between benign and malignant lesions. Chen et al. [95] investigated 42 patients with 71 SMs with or without vertebral compression fracture and plotted their MRI enhancement pattern over time. They classified “time–intensity curves” (TIC) into five classes (Figure 3).

They concluded that TIC type D predicted malignancy (with or without fracture) with a positive predictive value of 100%, and TIC type E was indicative of benign vertebral compression fracture with a positive predictive value of 86%. A secondary plateau in TIC type C (which reflects gadolinium equilibrium between intra- and extravascular spaces) was seen in acute, chronic, benign, and malignant lesions/fractures and had no significant predictive value. They also found that semi-quantitative peak enhancement percentage and wash-in slope can differentiate malignant fractures from chronic benign fractures, but in acute fractures, neither TIC type nor quantitative parameters could differ benign from malignant processes.

For quantitative analysis, the commonly used Tofts two-compartment model assumes contrast to be present only in the intravascular and interstitial spaces [96]. Lesional contrast in the intravascular space gives estimates of Vp, the plasma volume fraction which provides an estimate of the tumor vascularity, Ve; the extravascular extracellular contrast fraction; and K^trans^, a measure of s capillary permeability [97].

**Figure 4:**
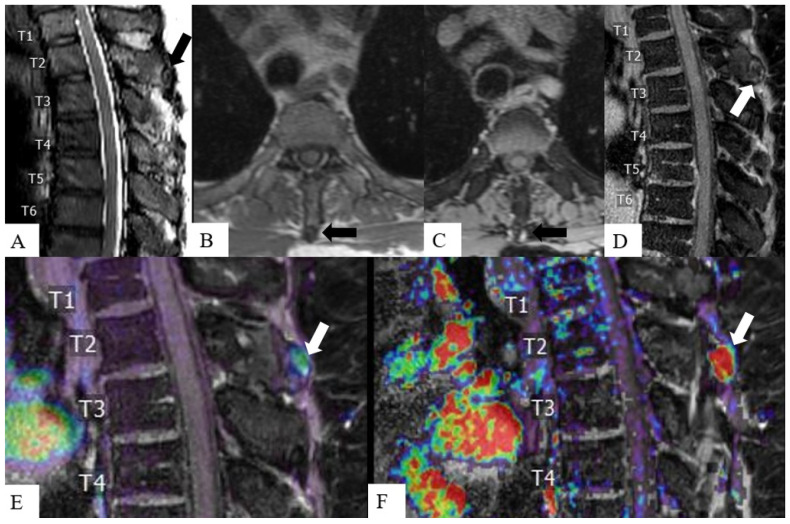
Metastatic lesion in the spinous process of the T2 vertebra. The sagittal T2-weighted image (**A**) displays a subtle oval-shaped lesion (arrow) involving the tip of the T2 spinous process. On pre-contrast axial T1-weighted image (**B**), the lesion shows low signal (black arrow), and peripheral enhancement on axial (black arrow) (**C**) and sagittal (white arrow) (**D**) post-contrast T1-weighted images. Post-contrast T1-weighted images overlaid with dynamic contrast-enhanced parametric maps improve lesion visibility and detection compared to conventional MRI, showing increased Vp (**E**) and K-trans (**F**) at the lesion site (white arrows).

DCE microvascular and hemodynamics assessment may detect SMs earlier or make them more conspicuous compared to conventional MRI, which solely relies on morphological changes and water content of the vertebral body [98] (Figure 4). SBRT results in microvascular ablation in tumors [99], and given that DCE informs tumor vascularity, DCE may contribute to pre-SBRT planning [100,101] and post-SBRT follow-up.

**Figure 5:**
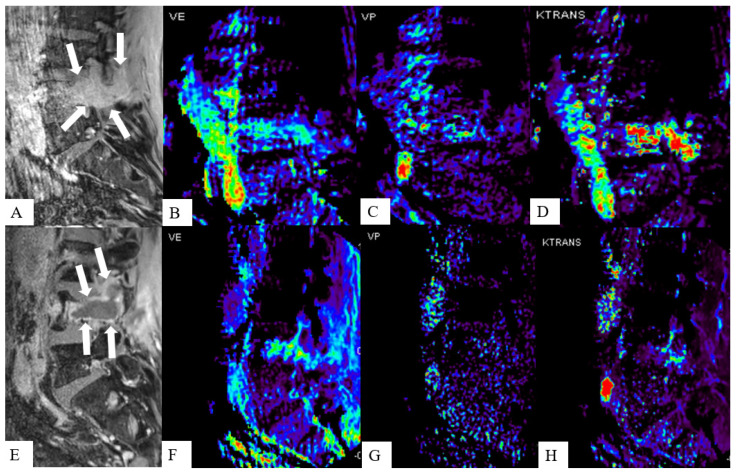
Monitoring response treatment in a 51-year-old male with spine metastasis from renal cell carcinoma. Pre-SBRT images (**A**–**D**) show a solid lesion with irregular margins and moderate enhancement on the sagittal post-contrast T1-weighted image (**A**) involving the vertebral body and pedicle (arrows). Ve (**B**), Vp (**C**), and K-trans (**D**) dynamic contrast-enhanced parametric maps show increased perfusion indices within the lesion. Post-SBRT imaging (**E**–**H**) shows considerable central necrotic change in the mentioned tumor 4 months after SBRT treatment (arrows). Notice the significant lesion perfusion loss depicted in Ve (**B**), Vp (**C**), and K-trans (**D**) parametric maps indicating treatment response, despite stable lesion size on conventional MRI, highlighting the ability of DCE perfusion to better represent metastatic lesion biological activity.

DCE has been shown to be effective not only in differentiating between normal and infiltrated marrow, but also in differentiating benign from malignant vertebral compression fractures [95,98,102,103,104]. A single-institution study by Kayhan et al. [105] investigated 16 patients with SMs from prostate adenocarcinoma and concluded that all the SMs showed early and substantial enhancement with a significantly higher K^trans^ and Ve than normal bone marrow 104. Khadem et al. [100] retrospectively investigated 26 SMs patients, and reported that DCE could differentiate hypervascular from hypovascular SMs and treated from untreated SMs using TIC patterns and semiquantitative parameters, with a higher accuracy than post-contrast T1-weighted MRI. Likewise, quantitative parameters can differentiate hypo- and hypervascular SMs [106]. Generally, hypervascular SMs show a higher peak enhancement, higher wash-in slope, and larger Vp and K^trans^ values. DCE’s ability to non-invasively identify tumor vascularity before treatment may improve targeted SM treatment, including consideration for tumor embolization [107]. Previous studies have demonstrated the earlier detectability of changes in vascular parameters on DCE-MRI compared to morphological alterations on conventional MRI in patients who have undergone either RT or systemic antiangiogenic treatment [92,108,109] (Figure 5). DCE may predict treatment failure 6 months earlier than conventional MRI [110]. Among the different DCE parameters, Vp maps showed the strongest predictability in confirming treatment success, with a Vp decrease predicting a good treatment response [92,93,110].

Studies have shown that DCE can differentiate local failure from treatment changes [111,112]; likewise, Chu et al. showed that longitudinal Vp decline is most strongly associated with treatment response, while Vp increase suggested LF [93]. The latter is most likely caused by neoangiogenesis which has been shown to be increased with recurrent bone metastasis [113]. Spratt et al. indicated that K^trans^ and Vp together can predict successful treatment response with 100% accuracy, and with better performance than monitoring morphological changes on conventional imaging or clinical symptoms [114].

Despite its advantages, due to its limited availability, restricted field of view, significant inter-institutional variability in acquisition protocol, the small sample size of the current supporting studies, the absence of established cut-offs for perfusion indices, and optimal timing for DCE-MR perfusion, the diagnostic utility of DCE MR perfusion is still under investigation [92].

### 8.2. DWI

Diffusion-weighted imaging (DWI) is an MRI technique that assesses the free motion of water molecules in tissue. DWI is increasingly being utilized for the spine; however, it faces technical challenges due to the small size of the cord, involuntary motion, and divergent susceptibility features in surrounding tissues.

To overcome these technical challenges and better incorporate DWI into spine MRI in patients with SMs, spine DWI typically uses a shorter echo time (TE) compared to brain DWI, employs a lower B value (400–600), and lowers the number of frequency-encoded steps. In addition, spine diffusion should be performed with higher resolution and homogenous fat saturation. For fat saturation, spectral-attenuated inversion recovery (SPAIR) performs well while maintaining a signal–noise ratio, while Dixon provides a very homogeneous fat suppression over large fields of view (FOVs) [115]. Another issue with spine DWI is the substantial anatomic distortion with single-shot echo-planar imaging, as the long readout accumulates phase errors that can cause spatial mismatch and blurring and its low bandwidth along the phase-encoding direction causes severe image distortion in areas of susceptibility inhomogeneity, which is especially problematic in spine due to complex anatomy and tissue interfaces 115. Multiple distortion reduction techniques have been discussed in the literature and implemented into clinical practice, including reduced FOV excitation, rectangular FOV using line scan diffusion imaging, interleaved EPI, propeller DWI, using readout-segmented EPI in readout or phase direction, or using isovolumetric signal collection to be able to choose transverse orientation [116,117,118]. These methods create a more rapid k-space traversal that decreases artifacts resulting from off-resonance spins [116]. Line scan diffusion imaging is not sensitive to magnetic susceptibilities and inhomogeneities and can build a rectangular FOV with a series of columnar single shot acquisitions [117].

DWI adds microstructural information to conventional imaging in addition to tumor cellularity assessment [119]. A higher nuclear–cytoplasmic ratio in tumoral cells and increased density of cells results in the restriction of intracellular and extracellular fluid motion, leading to increased DWI signal.

DWI can increase the conspicuity of SMs compared to conventional MRI sequences (with almost 10% increased lesion detection) [28,120,121,122,123]. DWI may also differentiate between benign and malignant VCFs [122,123,124,125,126]. DWI offers a better contrast–noise ratio when imaging non-sclerotic SMs compared to conventional MR sequences and higher sensitivity than CT or scintigraphy [127,128].

DWI may also capture treatment response failure earlier than conventional MRI [49,129], which could facilitate modifications in treatment protocols, leading to better patient outcomes.

With tumor response to treatment, the DWI signal decreases and ADC values increase [130,131]. Challenges to the utilization of DWI for SM treatment response evaluation exist, however, relating to technique limitations and susceptibility artifacts from spine hardware in the post-operative setting limiting image quality [92,132,133], and it remains an investigational technique at this time. While the existing studies are limited to DWI’s role in the post-EBRT setting, and to the best of our knowledge no research has yet explored its application in the post-SBRT setting, further investigation into this area is warranted.

## 9. Imaging with Spinal Hardware

With spinal hardware fixation, the spine may be obscured on MRI secondary to metal-related susceptibility artifacts. Accelerated dephasing within voxels leads to signal loss, and changes in local fields lead to spatial misregistration, causing geometric distortion, signal loss, or signal pile-up depending on the direction of spatial misregistration (in-plane or through-plane). Another issue with metal-related spatial misregistration and magnetic field inhomogeneity is poor fat suppression when spectral-based fat suppression techniques are used [134]. In this context, metal artifact reduction techniques, low-field MRI (1.5 T or lower), and CT myelography are the recommended alternatives [87]. Mitigating approaches include the acquisition of thinner slices, using turbo spin echo rather than gradient echo techniques, increasing the imaging matrix, keeping receiver and excitation bandwidths higher during slice selection and readout, and using shorter TE and echo spacing [135]. Specific advanced MRI sequences have also been developed and introduced during the last decade that can reduce signal distortion from magnetic field inhomogeneity caused by hardware [136,137,138].

CT myelography due to its invasive nature can lead to complications, including headache, infection, contrast allergic reactions, and neurological symptoms. CSF blockage can also limit subarachnoid space visualization above the level of obstruction [25].

Carbon fiber, often used with various resin matrices, is gaining traction in orthopedic surgery and oncology due to its advantages over metal fixation hardware. It is highly biocompatible, chemically inert, and non-toxic in vitro [139,140]. Carbon fiber is similar to bone in terms of its estimated elasticity, reducing stress at the bone–implant interface compared to metal [141,142]. Carbon fiber also shows promising durability, which improves fracture healing [143]. Carbon fiber also reduces beam hardening artifact on CT and susceptibility artifact on MRI, improving post-surgery image quality [144]. Unlike metallic implants, carbon fiber does not interfere with radiation planning or dose calculation, and improves radiation delivery [145,146,147] without increased rates of post-operative complications or hardware failure [148].

## 10. Treatment Response Assessment, Osseous Pseudoprogression and Post-SBRT Vertebral Compression Fractures

The ablative doses of radiation to SMs not only kill tumor cells, but also alter the microenvironment and microvascularity in adjacent normal tissues. Despite the fact that numerous studies have been conducted on response assessment after SBRT, most of these investigations focused on clinical response (specifically pain response which is the most important treatment goal in such cases) and very few studies have evaluated imaging response [92,149,150]. Post-SBRT imaging follow-up can evaluate epidural and SM disease evolution, altered tumor vascularity, and detect the development of new VCFs.

Criteria on Response Evaluation Criteria in Solid Tumors (RECIST) 1.1 guidelines assess treatment response in a quantitative manner measuring gross changes in the anatomical size (sum of the maximum diameter) of target lesion(s), but are applicable mainly to soft tissue tumors and does not generalize to most of the lytic and all of the blastic bony tumors [151,152]. They are also limited by the complex shapes typical of SMs. The SPINO group has recommended an alternative assessment method for assessing changes in GTV size in follow-up exams [48]. The main challenge with assessing response treatment based on RECIST 1.1 is that with slice thicknesses of 3 to 4 mm, slice selection may result in the imaging of tumors at different levels on successive studies [153], limiting inter-scan comparison and potentially presenting measurement inaccuracies. Three-dimensional imaging, however, can overcome this challenge, but is not always performed in post-SBRT follow-up. The SPINO group’s concept of “unequivocal increase” in GTV size also remains imprecise.

Osseous pseudoprogression (OPP) has been reported in 14–18% of the post-SBRT spine imaging cases [154,155]. OPP reflects imaging changes within the irradiated bone that can appear similar to tumor progression. It can appear as high T2-W signal intensity and can show post-contrast enhancement in the first few months after SBRT, which persists for 3 months and then shrinks in size [154]. OPP is typically asymptomatic and eventually regresses or stabilizes on imaging [59]. Local failure typically involves the epidural space, while OPP tends to remain confined within the vertebral body. OPP mostly shows a fast progression in size (within 5 months), is limited to the region of >80% isodose irradiation, and has a lytic appearance [81,155]. Exceptions occur, however, as with four reported cases of epidural OPP [155,156]. When imaging indicates that OPP is more likely than LF, histopathological evaluation is advocated by the SPINO group for confirmation [48].

Despite the absence of literature on the diffusion- or perfusion-weighted imaging of OPP in spine SBRT, advanced MRI techniques such as DWI and perfusion imaging can offer significant potential in distinguishing between pseudoprogression and true progression. In DWI, tumors responding to treatment tend to show an increase in ADC values even if conventional imaging does not confirm a decrease in tumor size. Furthermore, the presence of the foci of early and rapidly progressing enhancement in perfusion imaging post-treatment suggests tumor advancement, while the combination of a lack of early enhancement in perfusion images or tumor enlargement in conventional imaging highlights the low probability of true progression [157].

Post-SBRT VCF significantly decreases the quality of life and Karnofsky performance status score and increases narcotic usage from 40% to 70% [158,159]. VCF is the most common adverse effect of spine SBRT and occurs in 11–39% of the patients depending on radiation dose and fractionation scheme among other factors [149]. Some morphologic features on MRI can differentiate pathologic VCFs from benign fractures, but have limited accuracy [98,160]. A horizontal T1 and T2 low signal line (a band of trabecular compaction) or a true fracture line with adjacent edema with or without fluid or gas in the fracture cleft are compatible with benign fractures. Posterior endplate bulging (the convexity of the posterior cortex of the vertebral body), posterior element marrow SI changes (especially the pedicles), associated paraspinal masses, and bilobed ventral epidural masses favor pathologic VCF. Monitoring morphological changes on follow-up MRI can also distinguish benign VCFs from malignant fractures, as with the former marrow edema decreases over time, while in the latter, fluid SI in the marrow persists [49].

Nonetheless, conventional imaging modalities (CT or MRI) struggle to distinguish between therapy-related and LF-related VCF when analyzed based on individual imaging features, achieving a sensitivity of 26–86% and specificity of 79–99% for different CT or MRI findings [161,162]. By combining various imaging features and incorporating functional and advanced imaging, the accuracy of discrimination significantly improves [162,163]. Histopathologic evaluation reduces uncertainty and has been recommended by the SPINO group in the selected cases [48]. Advanced and functional imaging may be able to differentiate post-SBRT therapy-related and local failure-related fracture with higher accuracy and at an earlier stage [164]; however, they have not been well studied in post-SBRT settings. While advanced imaging offers several benefits, it also has limitations, and interpretation should be approached cautiously within the clinical context of each specific case. PET/CT should show a higher tracer uptake in malignant post-SBRT VCFs, but post-therapy local inflammation can also increase tracer uptake up to 6 months after SBRT [150,161]. In addition, PET/CT is less sensitive to osteoblastic SMs which show a lower affinity for metabolic tracers [48,165]. DCE MRI shows a steeper enhancement slope and greater peak enhancement percentage for acute VCF compared to chronic compression fractures but does not differentiate well between malignant post-SBRT VCF and benign acute VCF [95].

## 11. Future Directions

Imaging approaches to better localize the spinal cord for SBRT treatment planning can help reduce spinal cord radiation and the associated biological effects. A recent study found that physiological spinal cord motion detection using dynamic MRI and incorporation into SBRT treatment planning leads to reduced spinal cord doses in most patients. Interindividual variation in dose was reported to be wide (0.6–13.8%). The incorporation of dynamic MRI into clinical protocols, similar to our practice where it is performed in every SBRT planning, MRI could provide valuable treatment guidance to control spinal cord dose [166].

Traditional CT scanners use energy-integrating detectors, while photon-counting detector (PCD) CT relies on smaller detector pixels capable of counting individual photons and distinguishing their energy. At the same radiation dose, PCD CT provides significantly higher spatial resolution, resulting in less noise and clearer imaging of tumor margins and bone structures [167]. PCD CT could improve the visualization of small SM otherwise not detectable with conventional CT spatial resolution [168]. In terms of pre-treatment and post-treatment imaging, material composition using dual-energy CT and its ability to image or subtract calcium may prove beneficial for the detection and longitudinal evaluation of SMs [169,170].

## 12. Conclusions

Spine SBRT delivers high radiation doses to tumors with steep dose gradients which allow the sparing of the spinal cord and cauda equina, which are in the immediate vicinity of the target volume. The steep dose gradients require accurate radiotherapy planning and OAR delineation with high-quality imaging. Treatment-guided imaging equipment and novel surgical hardware have further advanced treatments and improved patient outcomes. Conventional CT and MRI, while providing valuable information, have limitations in the pre- and post-SBRT environment. Advanced imaging applications, including DCE perfusion and diffusion MRI, photon-counting CT, and dual-energy CT have the potential to better detect spinal metastatic disease, more accurately represent biological tumor activity compared to conventional imaging modalities, and potentially predict treatment complications and treatment failure. Techniques such as dynamic MRI may be able to better indicate spinal cord position to reduce potential cord dosing. Further investigation of advanced imaging applications is still needed, however, to better elucidate applications, value, and limitations.

## Figures and Tables

**Table 1:** SINS scoring system.

	SINS Criteria	Score
Pain	No pain	0
	Non-mechanical pain	1
	Mechanical pain	3
Spinal segment involved	Rigid (S2–S5)	0
	Semi-rigid (T3–T10)	1
	Flexible (C3–C6 and L2–L4)	2
	Junctional (Occiput–C2, C7–T2, T11–L1, and L5–S1)	3
Density	Blastic	0
	Lytic and blastic	1
	Lytic	2
Vertebral involvement	<50% involvement, no collapse	0
	>50% involvement, no collapse	1
	<50% collapse	2
	>50% collapse	3
Posterolateral involvement	Unilateral	1
	Bilateral	3
Spinal alignment	Normal	0
	Kyphosis or scoliosis	2
	Subluxation or translation	4

SINS, Spinal Instability Neoplastic Score.
